# Information and Communication Technology Based Integrated Care for Older Adults: A Scoping Review

**DOI:** 10.5334/ijic.6979

**Published:** 2023-04-03

**Authors:** Tian Yutong, Zhang Yan, Cheng Qingyun, Meng Lixue, Gao Mengke, Wang Shanshan

**Affiliations:** 1 The School of Nursing and Health, Zhengzhou University, Zhengzhou, Henan Province, China; 2 School of Nursing, The Hong Kong Polytechnic University, Hong Kong, China

**Keywords:** integrated care, information and communication technology, older adults, scoping review

## Abstract

**Background:**

Integrated care is an important initiative to respond positively to the ageing of society and information and communication technology(ICT) plays an important role in facilitating the integration of functional and normative health and social care. The scoping review aims to synthesize evidence on the experience and practice of ICT-based implementation of integrated care for older adults.

**Methods:**

This study followed the research framework developed by Arksey and O’malley for the scoping review and systematically searched for relevant studies published between 1 January 2000 and 30 March 2022 from nine electronic databases, three specialist journals, three key institutional websites, 11 integrated care project websites, google scholar and references of the studies to be included. Two reviewers independently screened and extracted data and used thematic analysis to sort out and summarize the core elements, hindrances and facilitators of ICT-based integrated care.

**Results:**

A total of 77 studies were included in this study, including 36 ICT-based practice models of integrated care with seven core elements of implementation including single entry point, comprehensive geriatric assessment, personalized care planning, multidisciplinary case conferences, coordinated care, case management and patient empowerment, which generally had a positive effect on improving quality of life, caregiver burden and primary care resource utilization for older adults, but effectiveness evaluations remained Heterogeneity exists. The barriers and facilitators to ICT-based implementation of integrated care were grouped into four themes: demand-side factors, provider factors, technology factors and system factors.

**Conclusion:**

The implementation of ICT-based integrated care for the elderly is expected to improve the health status of both the supply and demand of services, but there is still a need to strengthen the supply of human resources, team training and collaboration, ICT systems and financial support in order to promote the wider use of ICT in integrated care.

## Introduction

The world’s population is ageing in an increasingly serious way. In 2019, the global population aged 65 and over has reached 703 million and is expected to exceed 1.5 billion in 2050, and the number of people aged 80 or over will increase from 143 million in 2019 to 426 million in 2050, of which more than 50% will live in East and Southeast Asia [1]. The rapid increase in the proportion of the elderly population has put enormous financial pressure on the national system of elderly service provision. In addition, as older adults age, they are at increased risk of physical and mental decline, with a progressive increase in the prevalence of mobility loss, cognitive decline, hearing impairment and visual impairment, and increasingly complex health and social care needs. However, the World Health Organization (WHO) forecasts a global shortage of 18 million health care workers by 2030, particularly in Africa and South East Asia [2], posing the challenge of maintaining a balance between demand and supply of services for the elderly and the urgent need to find accessible channels to integrate medical and social resources to proactively address the ageing of society.

The United Nations Decade of Healthy Ageing (2020–2030) specifically identifies the development of integrated care as one of the areas of action to ensure that older adults have access to quality basic health services without discrimination [3]. Integrated care refers to the management and provision of services to provide people with continuous health promotion, disease prevention, diagnosis, treatment, disease management, rehabilitation guidance and palliative care throughout their lives and to coordinate care at different levels and locations both within and outside the health sector [4], with the aim of improving the inability of low-quality, inefficient elderly service provision to meet the increasingly complex healthcare needs of older adults. As the ‘lubricant’ and ‘glue’ of integrated care systems, the effective use of ICT can increase access to and flow of information, increase work efficiency, improve care integration and management processes, address COVID-19 concerns and social isolation [5], and has been identified as an important enabler of integrated care delivery and coordination of primary health care [6,7].

The World Health Organization has developed the Integrated Person-Centered Health Services (IPCHS) framework and the Integrated Care for Older adults (ICOPE) program, and has developed the ICOPE Handbook application to promote integrated person-centered services based on digital technology [8]. However, most integrated care programs for frail older adults don’t follow all WHO-IPCHS strategies and their clinical practice continues to suffer from inadequate resources and support, lack of coordination and interprofessional collaboration, and poor quality of person-centered care [9]. In addition, the adoption of ICT in community-based geriatric care has been slow, and its implementation in integrated care for older adults is often unsatisfactory due to policy, funding and infrastructure factors, with heterogeneity in clinical practice effects [10], and existing studies don’t provide an overview of the current state of implementation of ICT-based integrated care for older adults. To fill this gap in the evidence base, this study aims to provide an in-depth analysis and synthesis of the practice models, initial effects, potential barriers and facilitators of ICT-based integrated care for older adults using a scoping review approach, and to draw out policy opportunities and lessons that can be applied to the Chinese context.

## Methods

This study was conducted following the framework of a scoping review developed by Arksey and O’malley [11] and further updated by Levac et al [12], and the study protocol has been published in the BMJ Open [13]. We followed the JBI evidence synthesis manual [14] and the PRISMA-ScR checklist [15] to report the scoping review results.

### Stage 1: Identifying the Research Question

The aim of this study was to summarize the available evidence on the practice models, initial effects, facilitators and hindrances of ICT-based integrated care for older adults. With this research aim in mind, an initial search of the PubMed database was conducted and literature related to the research topic was read. Based on the researcher’s initial understanding of the current state of research, a refined research question was formulated following the PCC principles (population, concept, context) [16] as follows.

1. What are the service providers involved in ICT-based integrated care for older adults? What does the ICT used include and what is the functional role it plays?
2. What are the components of ICT-based integrated care services? What are the practice pathways and initial effects?
3. What are the hindrances and facilitators of ICT-based practice of integrated care?

### Stage 2: Identifying Relevant Studies

A systematic search of studies published between 1 January 2000 and 30 March 2022 was conducted using a combination of subject headings and entry terms, including “information and communication technology”, “Delivery of Health Care, Integrated” and “Aged”. Detailed search formulas for each database can be found in the supplementary materials. The search strategy was developed with the advice and assistance of experienced librarians. We searched a total of nine databases, Pubmed, Web of Science, EBSCO, Scopus, MEDLINE, EMBASE, CINAHL, Cochrane Library, Joanna Briggs Institute, and three specialist journals, International Journal of Integrated Care, Journal of Integrated Care and International Journal of Care Coordination. In addition, the official websites of three key agencies, the World Health Organization, International Foundation for Integrated Care, and European Commission, as well as the 11 integrated care projects websites of CareWell, BeyondSilos, Smartcare, SUSTAIN, CONNECARE, INTEGRATE, ValueCare, PROCare4Life, ProACT, SELFIE, and INSPIRE were searched, and the references of the proposed included studies and google scholar were manually searched to ensure comprehensiveness of the included studies. The retrieved studies were imported separately into the EndNote X9 literature management software and the search time for each database was recorded. An initial check was carried out by the reviewer based on the three main pieces of literature information: author, year and title, and duplicates were removed.

### Stage 3: Study Selection

Title, abstract and full text screening were conducted by two reviewers (TYT and CQY) based on study inclusion and exclusion criteria. Inclusion criteria: (1)the intervention/target/service population is older adults aged 60 and above; (2)the study describes and/or evaluates ICT-based practice models of integrated care, in which the integrated care need to follow the principles of comprehensiveness, multidisciplinary, and person-centeredness. ICT refers to the various technological tools and resources used to collect, store, retrieve, create, share or transmit information, including computers, the internet, live broadcast technology, recorded broadcast technology and telephony, etc.; (3)the literature is applicable to any type of health care setting, including primary health care, hospitals, emergency departments or medical consortia; (4)quantitative (intervention research, descriptive research, interpretation-prediction-correlation research), qualitative (phenomenology, grounded theory, action research) or mixed-method research designs are used; (5)the language of the paper is English. Exclusion criteria: (1)study of nonhuman subjects; (2)reviews, editorials and descriptive articles that do not provide relevant empirical evidence; (3)literature featuring no access to the full text or incomplete information. Two reviewers (TYT and CQY) searched and reviewed independently, and when there was disagreement about the inclusion of studies, a third reviewer (MLX) was consulted or discussed by the study team for a final decision.

### Stage 4: Charting The Data

The research team developed the data extraction checklist based on the research questions and the principles of person-centered, comprehensive and multidisciplinary nature of integrated care, specifically author, year, publication name, study title, country, study design, research questions/objectives, participant characteristics/sample size, model practice approach, and model practice evaluation. Tow reviewers (TYT and CQY) worked independently on the data using a data extraction form, and any disagreements were resolved through discussion in team meetings until consensus was reached.

### Stage 5: Collating, Summarizing, and Reporting Results

This study used quantitative (descriptive statistical analysis, frequency) and qualitative (descriptive content analysis) methods to analyze the scope, nature and distribution of the included studies and used graphical techniques to iteratively synthesize and interpret the findings by screening and ranking the material. Two researchers (TYT and CQY) followed Braun and Clarke’s thematic analysis to sort out and summarize the core elements, barriers and facilitators of integrated care content and used Nvivo software to code and analyze the data.

## Results

**Figure 1:**
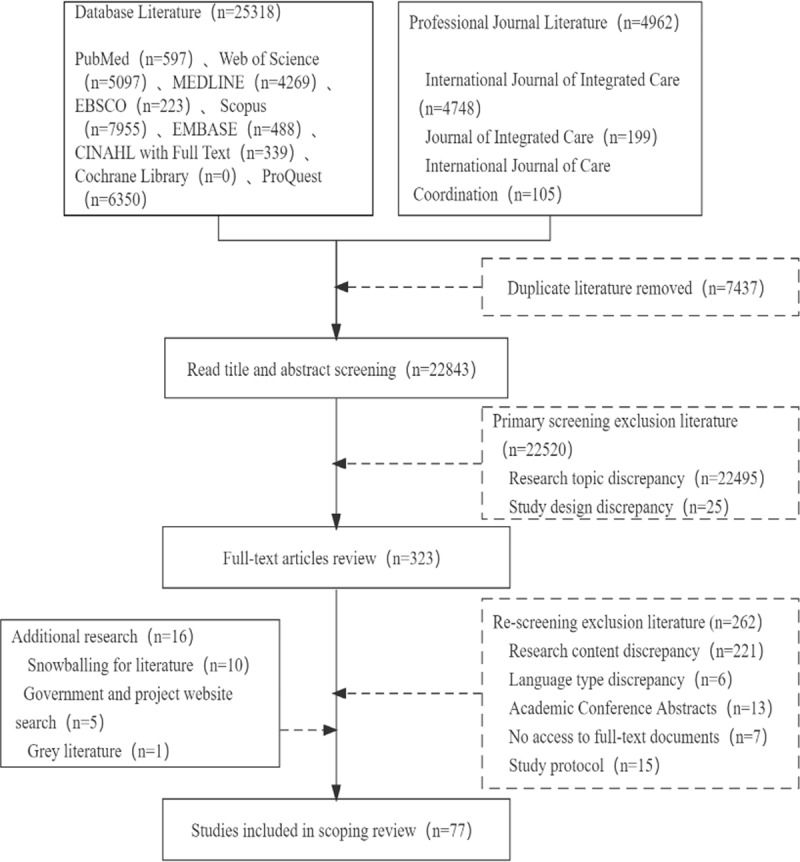
Flow chart of literature screening.

A total of 30,280 articles were retrieved in this study, of which 25,318 were from electronic databases and 4,962 from three professional journals, and 7,437 duplicates were excluded after preliminary check. After reading the titles and abstracts to exclude literature 22,520 articles, 322 articles were re-screened by reading the full text and further searched for their references, grey literature databases, government and project websites for additions, resulting in 77 studies being included in the review, of which 71 were published, one was grey literature and five were working papers. A flow chart of the screening process is detailed in Figure 1.

### Characteristics of Included Studies

**Figure 2:**
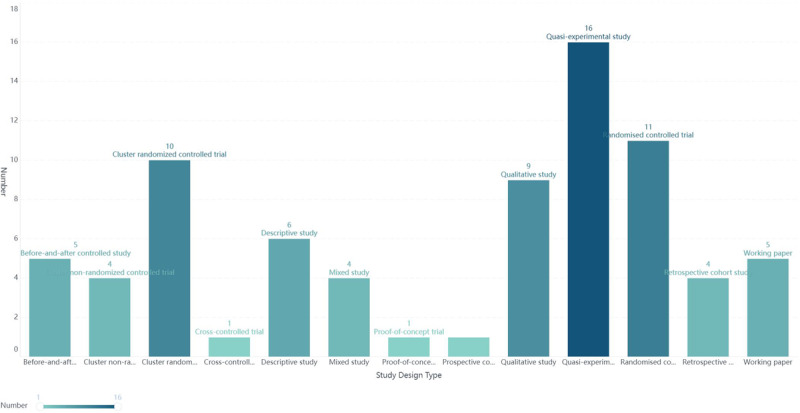
Distribution of study design types.

**Figure 3:**
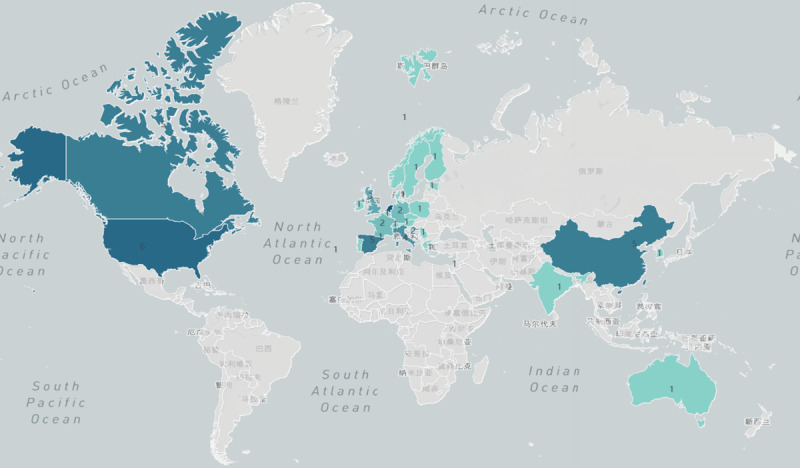
Regional map of the study distribution.

Of the 77 studies included, a total of 36 ICT-based integrated care practice models were included, of which only eight explicitly specified the rationale for their construction [17,18,19,20,21,22,23,24], with the Chronic Care Model (CCM) being used six times [17,18,19,20,21,22]. The 77 studies included 16 quasi-experimental studies [21,25,26,27,28,29,30,31,32,33,34,35,36,37,38,39], 11 randomized controlled trials [19,40,41,42,43,44,45,46,47,48,49], 10 cluster randomized controlled trials [17,24,50,51,52,53,54,55,56,57], 6 descriptive studies [58,59,60,61,62,63], 9 qualitative studies [18,23,64,65,66,67,68,69,70], 4 retrospective cohort studies [71,72,73,74], 5 working papers [75,76,77,78,79], 5 before-and-after controlled studies [80,81,82,83,84], 4 cluster non-randomized controlled trials [85,86,87,88], 4 mixed studies [20,22,89,90], 1 proof-of-concept trial [91], 1 prospective cohort study [92] and 1 cross-controlled trial [93], 60.5% of the literature was interventional studies, published in the time range 2003 to 2022 and the specific study design types are shown in Figure 2. The 36 practice models were implemented in 29 countries in Europe (22/75.9 %), Asia (4/13.8 %), North America (2/6.9 %) and Oceania (1/3.4 %), and the detailed distribution is shown in Figure 3.(http://localhost:37799/webroot/decision/link/Abp0). Integration of care was mainly for frail older adults (30.6%), older adults with physical or cognitive impairment (16.7%), older adults with multiple morbidities (13.9%), older adults with chronic conditions (13.9%), ordinary elderly (13.9%), older adults with complex needs (8.3%) and older adults with low income (2.8%). The characteristics of the included articles are detailed in Table 1.

**Table 1:** Characteristics of included studies.


MODEL NAME	COUNTRY	PRACTICE SETTING	TARGET GROUP	MULTIDISCIPLINARY TEAM MEMBERS	ICT	PRACTICE PATH	SERVICE CONTENT	MAIN FINDINGS

PRISMA (a Coordination-Type Integrated Service Delivery System) [25,26,27]	Canada	Home	Frail elderly	Medical specialist, physiotherapist, occupational therapist, speech therapist, primary care physician, case managers (nurses, social workers or other health professionals, etc.)	Telephone, case-mix classification system, geriatric information system	Coordination of decision makers and managers at regional and local levels; Single entry point; Single assessment tool combined with a mixed case management system; Case management; Development and regular review of individualized service plans; Computerized clinical charting.	Home delivered meals, day center institutionalization, elderly care, professional care and rehabilitation services, therapy, home care, etc.	Preventing functional decline. Meeting the needs of elderly. Satisfaction and empowerment rates improved; Reduced caregiver burden. Improved utilization of healthcare services. The number of emergency room and inpatient admissions was lower than expected.

Bois-Francs Integrated Service Delivery (ISD) Network [28]	Canada	Community health center	Frail elderly	Health care professional, primary care physician, case manager	Geriatric Information System	Interdepartmental coordination at strategic, tactical and clinical levels; Single entry point; Single patient assessment tool; Case management; Development of personalized service plans; Computerized clinical charting.	Specialist medical services, home care, day care, rehabilitation, elderly care, primary care services, etc.	Reduced hospitalization rates and willingness to stay. Reduced caregiver burden. Delays the decline in function and deterioration of frail older adults in the short term. A smaller proportion of emergency visits resume within 10 days of the first visit. No significant difference in service utilization, emergency care, hospital admissions or medication use.

Transitional Care of Older Adults Hospitalized with Heart Failure [40]	United States	Hospital and home	Mental Failure Elderly	3 Advanced practice nurses (APN), doctor	Telephone, tape and recorder audio material	1.Hospitalization: comprehensive patient assessment, identification of patient and caregiver health goals, development and implementation of individualized care plans (guided by guidelines), provision of educational and behavioral strategies, arrangement of needed home care services, coordination with discharge planners for ordering of essential medical supplies. 2. After discharge home: targeted assessment to identify changes in the patient’s health status, implementation of symptom prevention or impact reduction strategies.	Specialist medical services, telephone follow-up, health education, home care, etc.	Extended the time between discharge and readmission or death. Decreased the total number of readmissions. Reduced medical costs.

Geriatric Resources for Assessment and Care of Elders (GRACE) [41,42,58]	United States	Hospital and primary care center	Low-income older adults	GRACE Support Team (1 Advanced practice nurse and 1 Social worker), Primary care practitioner, Geriatrician, Pharmacist, Physiotherapist, Mental health practitioner and Community services liaison	Electronic medical record, Regenstrief medical record system, telephone	In-home comprehensive geriatric assessment; Development of individualized care plans; Activation of GRACE protocols and team recommendations (based on practice guidelines); Review, revision and prioritization of care plans; Implementation of care plans; Weekly GRACE interdisciplinary team meetings; Care management and coordination of care; Telephone or face-to-face follow-up; Proactive follow-up of plans and provision of required health education materials.	12 GRACE intervention programs (advance care planning, health maintenance, medication management, walking difficulties/falls, chronic pain, urinary incontinence, depression, malnutrition/weight loss, visual impairment, hearing loss, dementia and caregiver burden)	Reduced costs for high-risk patients. Improved quality of care for high-risk populations. Reduced acute care utilization. Better acceptance by patients and their primary care physicians and feasibility of the program.

Guided care [21,29,50,51,52,53,54,55,56]	United States	General practitioner (GP) clinic	Older adults at high risk of chronic disease	Guided Care Nurse (GCN), Primary Care Doctor	Telephone, electronic health record	In-home assessment of patients and primary caregivers; Determine the priority of optimizing health and quality of life; Development of evidence-based care plans; Promotion of patient self-management; Monthly telephone monitoring of patient conditions and actions; Coaching of patients in practicing health behaviors; Coordination of care; Education and support of caregivers; Referrals to accessible community resources.	Specialist medical services, evidence-based care, active monitoring, transitional care, self-management guidance, caregiver support, community services, etc.	Improved primary care experience and problem-solving skills for older adults at high risk of chronic disease. Increased satisfaction with care from primary care providers. Reduced use of hospital care, professional care, rehabilitation facilities, home health care and acute care. Has some service economy.

Multidisciplinary integrated care model [57]	Netherlands	Residential care facility	Older adults with physical or cognitive disabilities	Nurse assistant, family doctor, consultant (geriatrician or psychologist)	Electronic Integrated Geriatric Assessment Tool	Multidimensional geriatric assessment every three months; Discussion of assessment results and care priorities with family doctor, older adults and their families; Development of individualized care plans; Multidisciplinary team meeting; Geriatric or psychological specialist consultation (frail older adults with complex medical problems); Adjustment of care plans every three months based on risk assessment reports for older adults.	Support for activities of daily living, specialist care (medication guidance, wound care), home help, medication supervision, psychological counselling, etc.	Improved the quality of residential institutional care for older adults. A reduction in the number of deaths. No significant impact on improving functional capacity, number of hospital admissions and health-related quality of life.

Primary Integrated Interdisciplinary Elder Care at Home (PIECH) [80]	Canada	Home	Frail elderly	Community nurse, primary care physician, physiotherapist, doctor, nurse	Telephone, email, fax, electronic health record	1.Primary care: comprehensive geriatric assessment, discussion and documentation of health care instructions, division of labor among team members in the provision of services. 2.Hospital care: hospital treatment, sharing of personal health records, clinical case management, provision of supportive care and assistance with discharge planning.	Case management, primary health care, specialist medical services (cryotherapy, joint injections, physiotherapy, bowel and bladder care, wound care), telephone consultations, home support, etc.	Reduced acute hospital admissions and facilitated family deaths.

Coordinated-Transitional Care (C-TraC) program [71,72,81]	United States	Hospital and home	Older veterans (with CHF and COPD)	C-TraC Nurse, Case Manager (Nurse)	Telephone, electronic medical record	Identification of eligible participants based on multidisciplinary discharge visits; Inpatient visits (to discuss medication management, post-discharge medical follow-up plan, red flags, contact information, etc.); Post-discharge telephone follow-up (to perform medication reconciliation, risk signal assessment, ensure appropriate follow-up, provide education, etc.)	Case management, palliative care, outpatient care, geriatrics and telemedicine, disease deterioration and coping education, medication management, etc.	Good fidelity of C-TraC program implementation. Reduced 30-day readmission rates for veterans. Saved an average of $1,842.52 per person in medical costs with lower operating costs and resources.

CareWell in Hospital program [82]	Netherlands	Hospital	Frail elderly surgical patients	CareWell team (1 geriatric care specialist and 1 geriatrician), nurse, doctor, volunteer team	Clinician and patient information system, nurse information system	Initial frailty screening and clinical judgement of admitted patients; Critical assessment of patient medical information and medication use; Proxy medical records; Comprehensive geriatric assessment; Multidisciplinary meetings; Development of CareWell plan; Follow-up during admission; Update of CareWell plan at discharge.	Specialist medical services, medication, end-of-life care, volunteer support, etc.	Improved the elderly’s ability to perform activities of daily living between hospital discharge and follow-up. Reduced caregiver burden 3 months after discharge from hospital.

Walcheren Integrated Care Model (WICM) [30,31,32,33,34,39]	Netherlands	GP clinic	Frail elderly	GP, community nurse, hospital geriatrician, nursing home doctor, physiotherapist, social worker or psychologist, case managers (single-needs older adults: geriatric nurse; multiple or complex-needs older adults: second-line geriatric care specialist)	Patient file sharing system, telephone	Screening for frailty in older adults; Single entry point (primary care); Evidence-based comprehensive needs assessment; Development of multidisciplinary personalized service plans; Case management; Multidisciplinary team consultation and meeting; Protocol-led care assignment; Formation of steering groups (responsible for planning and implementing interventions); Task specialization and delegation; Chained computerized systems.	Telephone consultation, home visit, medical service, nursing home service, home care, day care, complementary medicine (physiotherapy, occupational therapy, nutrition), psychological care, informal caregiver support, etc.	Reduces the subjective burden on informal caregivers and increased possibility of assisting in housework. Improved the attachment dimension of quality of life for older adults. No significant impact on older adults’ health status, service provider workload and satisfaction and informal caregiver satisfaction with caregiving. WICM was not cost effective, with a higher cost per quality-adjusted life year.

Embrace integrated care program [19,43,44,64,83,84]	Netherlands	GP clinic	Older adults	GP, nursing home doctor, case managers (community nurse and social worker), volunteer	Clinical information system, electronic records system for the elderly	Complexity of care needs and frailty assessment for older adults (robust, frail, complex care need);Case managers develop care and support plans in consultation with participants (robust: self-management support and prevention plans; frail and complex care need: individual care and support plans); Setting health goals and taking action; Case managers monitor participant status and plan implementation; Holding regular Embrace community meeting; Regularly assess care and support plans, update and adjust as necessary.	Needs and vulnerability assessment, specialist medical service, disease surveillance, health education, etc.	Embrace counteracted the decline in physical, cognitive and social functioning associated with ageing. Improved quality of care. Higher overall mean costs and small, statistically insignificant differences in health-related outcomes.

Integrated Care and Discharge Support for elderly patients (ICDS) [92]	Hong Kong, China	Hospital and home	Older adults	Link nurse, geriatrician, case managers (2 social workers, 1 physiotherapist, 1 occupational therapist and half an advanced practice nurse on a rotating basis), doctor, pharmacist, etc.	Telephone	1. Hospital: multidimensional assessment of elderly patients, risk stratification, development of discharge plans, linkage to community services based on assessment results. 2. Community: Case management (out-of-hospital follow-up, coordination of community service, ensuring patient compliance with plans) and Home Support Team (HST) services.	Specialist medical services, case management, HST services (community support, meal delivery, home cleaning, respite care and home assessments and adaptations), etc.	Accident and emergency department visits, acute admissions and bed days have been reduced. ICDS had the potential to save on healthcare costs.

SmartCare program [75]	Austria, Croatia, Germany, Denmark, Estonia, Greece, Israel, Spain, Finland, Italy, Netherlands, Portugal, Serbia, United Kingdom, Czech Republic, Sweden	Hospital and home	Older adults	Nurse, GP, medical specialist, social worker, caregiver, third sector organization and volunteer	Electronic record system, electronic message, mail, fax, telephone, etc.	1. Integrated care pathways: integrated long-term home care support (two entry points: referral by health care provider, referral by social care provider), integrated post-discharge home support (single entry point: discharge from hospital impending). 2. Integrating Care Processes: assessment of care recipient’s needs for long-term/short-term home care, enrolment to SmartCare service, initial integrated care plan, coordination of integrated care delivery/revision of initial integrated care plan, personalized multi-provider service package, shared documentation of home care provided, monitoring/review/reassessment of care recipient’s needs, temporary admission to institution/disenrollment from SmartCare service	On-site/home provision of informal care, formal health care, social care, telecare, social care, etc.	Reduced the number and length of hospital stays for older adults. Increased the ability of older adults to self-manage their chronic conditions. Reduced hours of care for caregivers. Care costs were reduced, with a certain cost-effectiveness.

CareWell primary care program [85,86,87,88]	Netherlands	GP clinic	Frail elderly	GP, practice nurse and/or community nurse, geriatric nurse, pharmacist, social worker, case managers (nurse or social worker)	Health and welfare information portal (ZWIP), Electronic health record	Multidisciplinary team meetings (1 every 4–8 weeks); Proactive care planning (individualized care plans based on EASY-Care TOS assessment of individual health-related goals and needs); Case management; Medication reviews; Multidisciplinary practice guidelines, advance care planning practice guidelines for 8 common geriatric syndromes.	Medical, nursing and social support service, case management, medication guidance, etc.	No net monetary benefit. No significant impact on improving active functioning, quality of life, mental health, institutionalization, hospitalization and mortality in older adults; No observed effect on improving caregiver quality of life, caregiving burden.

Integrated care for geriatric frailty and sarcopenia [45]	Taiwan, China	Community hospital	Frailty and sarcopenia elderly	Nurse, sport specialist	Telephone, multimedia health education materials	1.Low level of care(LLC): Provide 2-hour educational sessions (frailty, muscle loss, coping strategies, nutrition and learning exercise program presentations); distribute multimedia health education materials; telephone follow-up visit. 2.High Level Care (HLC): 6 on-site problem-solving sessions and 48 exercise sessions on an LLC basis, with brief nutritional advice during exercise.	Health education, exercise, nutritional counseling, telephone follow-up, etc.	Improved frailty and muscle loss of community elderly. Higher levels of care improve to a greater extent for high risk and highly motivated older adults.

Integrated care at home [46]	Switzerland	Home	Frail and dependent elderly	Primary care physician, nurse, doctor, physiotherapist and occupational therapist, psychologist, nutritionist and social worker	Telephone	In-home assessment by the Community Geriatrics Unit (CGU); Recommendations from the primary care physician and care team based on the assessment; Multidisciplinary team meetings to discuss complex issues; Coordination of care (primary care physician or CGU providing a 24h medical call service).	Primary care and home visit nursing service, 24h medical call service, etc.	Reduced unnecessary hospital admissions, emergency visits. Improved care coordination and access to services for frail and dependent older adults.

Health TAPESTRY Integrated care approach [20,47,65,66]	Canada	Primary care clinic	Older adults	Family doctor, resident, nurse, pharmacist, various allied health professional, combination of volunteers (1 person with volunteer experience and 1 university student)	Health TAPESTRY application (TAP-App), electronic medical record, personal health record, telephone	Volunteer home visit (to discuss the health and life goals and needs of older adults); TAP-App based assessment data collection, creation of TAP reports; Review of TAP reports by volunteer coordinators; Regular interdisciplinary meetings to review reports; Development and implementation of personalized care plans; Community engagement and linkages.	Clinic visits, telephone consultations, specialist medical service, community service, etc.	Not improving patient goal attainment and reported outcomes. Increased the number of primary care visits for older adults. Reduced the incidence of 1 or more hospital admissions. Facilitated a shift from reactive to proactive and preventive care for patients. Initiatives to improve sustainability: team member engagement and training, clinical leadership involvement, infrastructure for sustainability.

Transitional care model for hospitalized cognitively impaired older adults [93]	United States	Hospitals and home/care facilities	Cognitively impaired elderly	APN, clinician, primary care provider	Telephone	Face-to-face assessment of patient and family caregiver needs and goals within 24 hours of admission; Inpatient visit; Design and implementation of care plans; Visitation and telephone services within 24 hours of discharge; Coordination of care (APN accompanies visits to primary care provider); Interdisciplinary team case review.	Specialist medical service, primary care service, telephone consultation, out-of-hospital follow-up, case management, etc.	Reduced the amount of post-acute care and the total cost of care.

Integrated Care for Older Adults with Complex Health Needs (iCOACH) [23]	Canada	Hospitals and community health centers/long-term care facilities	Elderly (complex health needs)	Doctor, nurse practitioner, nurse, pharmacist, community health worker	ICT system, electronic medical record	Institutional collaboration (supported by standardized referral procedures, single point of contact or shared assessment tools; clear division of responsibilities; mutual trust between providers); Holistic assessment of health and social care needs of older adults; Health education; Coordination of health and social services.	Home care, specialist medical service, primary health care, clinical assessment, health education, etc.	ICT application: community resource and policy, health system, delivery system, self-management support, decision support and clinical information system. Barriers to ICT use: barriers to cross-organizational access to information, lack of interoperability between organizational and regional systems, more limited application of IT functionality by providers.

INTESA integrated care project [59]	Italy	Nursing home	Frail elderly	Medical specialist, GP, physiotherapist, practice nurse, social health assistant, educator	DoEatWell App, INTESA subsystem, sleep monitoring sensor, pressure monitoring service mobile App	Personalized health indicator monitoring; Smart device-based collection, uploading and synchronization of monitoring data; Data analysis to calculate behavioral and physiological markers; Feedback and personalized guidance to GPs and caregivers.	Personalized monitoring service, 1h group cognitive and motor rehabilitation, 24h nursing home care service, social education activities, medical support (psychological, nutritional and neurological counselling), etc.	Perceived practicality, usability, acceptance and satisfaction of frail older adults was good. Some participants had a fear of adopting the INTESA equipment and implementing the recommendations and low self-confidence in completing the activities due to their frail health condition.

ICARE4EU project [60,61]	Italy	—	Multi-morbid elderly	——	Electronic health record (76%), personal health record platform (67%), digital communication between care provider (52%)	Of the 101 projects in Europe that integrate care for people with multiple morbidities, 31 use at least one eHealth technology and focus on people over 65 years of age.	Tele-consultation, monitoring and care; self-management, healthcare management, health data analysis (decision support)	Benefits of eHealth technology: care management, care integration, quality of care, cost efficiency, quality of life. Barriers to the use of eHealth technology: Lack of skills among providers, Inadequate technical ICT support, Lack of skills among patient, Inadequate legislative framework, Compatibility between different eHealth tool, Inadequate ICT infrastructures, Inadequate funding, Uncertainty of cost-efficiency, Privacy/security issues, Resistance by care providers, Cultural resistance, Resistance by patients

Salford Integrated Care Program (SICP)/Salford Together [76,89]	United Kingdom	Community health center	Older adults (long-term illness and social care needs)	GP, nurse, district nurse, social care worker, mental health practitioner, occupational therapist and administrator	Telephone, shared care record, electronic medical record	Integration of community assets; Mobilization of older residents for local service improvement; Formation of integrated contact center; Screening and risk stratification of older adults; Shared care protocol; Care plans based on patient risk and need; Care and support; Multidisciplinary case conference.	Specialist medical service, telemonitoring, telecare, health coaching, mental health, community service, etc.	SICP had economic cost-effectiveness. Improved quality of life for patients. Increased use of community assets and care plans and positive health coaching experiences.

Finding and Follow-up of Frail older persons (FFF) [22,35]	Netherlands	Home	Frail elderly	GP, practice nurse, family nurse or geriatric nurse, geriatric nurse practitioner, physiotherapist, case manager	Electronic medical record, GP information system, chain information system	Patient selection; Active frailty screening (reporting needs and problems); Feedback; Organization of multidisciplinary consultation; Creation of individualized care plans (lifestyle intervention, self-management measure, multidisciplinary follow-up and assessment plans); Medication review; Multidisciplinary follow-up.	Specialist medical service, home care, day care service, medication review, case management, self-management support, remote monitoring, etc.	Improved quality of care for frail older adults. FFF program was not cost effective. GPs saw structured funding and human, accessible ICT systems as key to the sustainable spread of FFF.

Comprehensive patient-centered strategy for multimorbid patients [73]	Spain	Hospital	Multi-morbid elderly	Liaison nurse, case manager, advanced skills nurse and internal medicine doctor	Electronic health record, electronic prescription	Development of chronic disease care plans for multidisciplinary teams; Coordination of care between different specialists during hospitalization; Telemedicine and empowerment services; Telecare services (coordination of care, sending health plan information or medication reminders, specialist training to address clinical and emotional problems).	Specialist medical service, telemedicine, telecare, telehealth education, primary care service, etc.	Reduced risk of admission to hospital for patients.

RESPOND (patient-centered program) [48]	Australia	Hospital and Home	Older adults attending A&E (falls)	3 physiotherapists, 2 occupational therapists, 1 nurse and 1 nutritionist	Telephone	In-home assessment of fall risk factors; Provision of four educational leaflet modules (strength, vision, sleep, bone) and evidence-based information on risk factor management; Encouragement of participants to select relevant modules; Development of individualized goals and action plans for each module; Identification and resolution of issues that prevent participants from implementing the program; Telephone support; Communication and coordination of community services.	Emergency service, fall prevention exercise, risk factor assessment, health education, community service, etc.	Improved prognosis for emergency patients. Reduced falls and fractures in patients, but no reduction in fall injuries in older adults.

ProACT Integrated Care Platform [67,91]	Belgium, Ireland	Home	Multi-morbid elderly	Informal care worker, formal/social care worker, community doctor, pharmacist and hospital doctor	Support CareApps, wearables, home-based sensor	Integration and coordination of care; Customization of the structure and functionality of CareApps based on the needs of older adults; Customization of data reports on the health and wellbeing of older adults using a color-coded traffic light system; Alerts and message pushing of abnormal values; Development of personalized health goals and self-management plans.	Telemonitoring, self-management education, clinical triage services, etc.	Older adults and stakeholders had a positive attitude towards the ProACT platform, with high perceived usability and benefits. Participation barriers: technical barriers (complex process of using devices and apps, lack of trust in the readings of smart monitoring devices), complexity of participants’ conditions.

CareWell integrated care model [36,37,77,78]	Spain, Croatia, Poland, Italy, United Kingdom	Hospital and home/health center	Multi-morbid complex elderly	GP, social worker, medical specialist, care manager, primary care nurse (PC)	Electronic health record, electronic health call center, personal health folder	Screening of frail elderly patients; Comprehensive baseline assessment; Multidisciplinary case conference; Development of individualized care plans; Integrated care during hospitalization and coordinated discharge; Programmed follow-up; Patient empowerment and home care (KronikOn).	Telemonitoring, telemedicine consultation, medication prescription, transition support (coordination of referrals), patient follow-up, health education, etc.	Improved information, coordination and participation, patient empowerment and family support in the care process. Reduced the number of emergency room visits Reduced length of stay in hospital. Reduced patients’ body mass index, blood oxygen saturation and blood glucose Changed the use of health resources and strengthened the key role of primary care. Positive or negative socio-economic returns depending on the region.

BeyondSilos (Telehealth-Enhanced Integrated Care Model) [38]	Spain	Home	Elderly with chronic comorbidities	Doctor, nurse, social worker, family worker, volunteer and third-party group, case manager	Home care platform, telephone	1.Care pathway: Integrated short-term family support following an acute episode; Integrated long-term family support. 2.Care processes: Ongoing assessment of older adults’ needs; Development and sharing of care plans; Single entry point(case manager); Regular visits or telephone contact with older adults; Ongoing follow-up of older adults’ health against care plans; Automatic alerts when health conditions deteriorate and accidents occur; Sharing of clinical information.	Specialist medical services (wound care, medication assistance, etc.), telemonitoring, telecare, home support (dietary and bathing support), volunteer accompaniment, etc.	Outstanding cost effectiveness. No significant effect on improving activities of daily living, depression.

Transitional care intervention for hypertension control [24]	China	Hospital and community health center	Geriatric diabetics	Medical specialist, GP, hospital discharge nurse, community nurse	Telephone	1.Health care systems: two-way referrals. 2.Service providers: personalized discharge education, development of individualized medication regimens, post-discharge support. 3.Individual level: hospital (setting goals; implementing plans;), home (acting to achieve self-care goals; monitoring and recording changes in health status in the “Patient Edition of the intervention diary”; regular visits to community health centers or telephone support).	Specialist medical service, self-management education, personalized medication, telephone support, primary care visit, etc.	Improved control of hypertension in older diabetic patients. Reduced readmission rates.

Integrated Care for Older adults, (ICOPE) [79]	China, Andorra, France, India	Community and primary care institution	Older adults (reduced intrinsic capacity)	Geriatrician, GP/primary care physician, resident, nurse, occupational therapist, physiotherapist, psychologist, pharmacist, health assistant, volunteer	ICOPE Handbook APP	Screening for areas of reduced intrinsic capacity; Person-centered assessment in primary care; Developing individualized care plans; Ensuring referral pathways and monitoring care plans (link to specialist geriatric care); Coordinating community involvement and supporting caregivers.	Specialist medical service, telemonitoring, multi-component intervention, primary care management, self-care and management, social care and support	Good identification and enthusiasm for ICOPE among older participants. ICOPE has encouraged coordination and collaboration between health and care workers. Facilitators: active participation of older adults, training of providers, digital integration of health information. Hindrances: human resources, lack of infrastructure and systems integration, financial barriers.

INSPIRE ICOPE-CARE program [62,63]	France	Hospital	Older adults	GP, nurse, physiotherapist, pharmacist, nutritionist, neuropsychologist and social worker	ICOPE MONITOR APP, BOTFRAIL internet conversation robot	Intrinsic capacity screening every 4–6 months; Person-centered assessment in primary care; Identification of care goals and development of individualized care plans; Ensuring referral pathways and monitoring of care plans with links to specialist geriatric care; Coordination of community involvement and support for caregivers.	Telemonitoring, telehealth consultation, telehealth education (nutrition, exercise), specialist medical service, etc.	The program had some feasibility. 70.4% of participants completed the 6-month follow-up screening 94.3% of older adults had a decline in at least one area of intrinsic ability.

Systems for Person-Centered Elder Care (SPEC) [17,90]	Korea	Nursing home	Frail elderly	Nursing home manager, nurse practitioner, social worker, physiotherapist, occupational therapist and nutritionist, SPEC coordinator (1 nurse, 1 social worker)	SPEC Information System, KaKao Talk APP	Integrated geriatric assessment (needs and risk analysis); Individualized care plans based on needs; Interdisciplinary case conferences; Coordination of care with family members, external health professionals and agencies; ICT support (sharing health assessment data, monitoring progress of interventions, providing information support).	Daily nursing home care, specialist medical service, etc.	SPEC plan had good fidelity. Improved the overall quality of care in nursing homes. Prevented deterioration of late ADL loss, cognitive and communication decline, new or persistent delirium and behavioral problems from occurring.

Personalized Connected Care (CONNECARE) project [49]	Spain	Home	Older adults with complex chronic conditions	Family doctor, hospital surgical team and social worker, case manager	Smart Adaptive Case Management System, Mobile Self-management System, Wearable monitoring devices	Initial assessment of patient health status; Generation of health status reports based on the self-management system; Customized virtual coaching with automatic feedback; Automatic tracking of patient activity; Shared patient profiles; Coordination of professional service providers; Case managers overseeing the entire care process.	Virtual coaching, nursing and social services, case management, etc.	Improved quality of life for patients. Reduced the number of unplanned visits and hospital admissions. Cost-effective savings of approximately €109.88 – €126.99 per patient.

Coordinated Care At Risk/Remote Elderly program (CCARRE) [68]	United States	Home	Cognitively impaired elderly	Bilingual/bicultural social worker, neurologist, primary care physician	Telephone, secure video platform	Telemedicine visits; comprehensive geriatric assessment; CCARRE medical review; Discussion of advance care planning, contingency planning and respite needs; Development of a comprehensive care plan for the patient’s cognitive status and presenting problems; Discussion of the CCARRE plan with the patient and caregivers; Shared plan reporting and coordination of care.	Teleassessment, telemedicine visit, health education, medication guidance, specialist medical service, respite service, etc.	Improved patient care. Reduced caregiver burden. Optimized access to community resources.

Person-Centered Care Through Videoconferencing [18]	Norway	Hospital and primary care institution	Older adults (with complex and long-term needs)	Doctor and medical secretary, nurse coordinator, geriatric nurse, physiotherapist and occupational therapist, pharmacist, case manager	Video conferencing platform, electronic health record system	Comprehensive geriatric assessment; Agreeing care goals with the patient; Developing an evidence-based care plan; Driving and implementing the care plan based on what is “important to the person”; Ongoing assessment and delivery of the care plan; Daily multidisciplinary team meetings.	Health care service, specialist medical service, medication guidance, etc.	Videoconferencing-based service delivery provided the opportunity for effective access to health care professionals. Reduced travel time for patients to access medical care. Improved information exchange between healthcare levels.

Integrated geriatric outpatient services (IGOS) [74]	Taiwan, China	Hospital	Multi-morbid elderly	Geriatrician, nurse, case manager	Telephone	Comprehensive geriatric assessment; interdisciplinary team care; person-centered care planning; single entry point (elderly patients with multiple complex care needs were primarily cared for by geriatricians)	Outpatient services, specialist medical services, case management, etc.	Improved quality of life for older adults with multiple morbidities. No significant impact on the improvement of quality of life for older adults with poorer nutritional status, depression and frailty.

RubiN (Continuous Care in Regional Networks) [69,70]	Germany	Primary care institution	Older adults	GP, specialist, nurse, physiotherapist, occupational therapist, healthcare assistant, 4 case managers	Geriatric Care Network	Risk assessment; Classification of patients as mild, moderate or severe cases according to their care requirements; Development of individualized and optimal treatment and/or care plans; Coordination and organization of medical care; Organization of case discussions and “round tables” to assess patient care.	Nurse support, social services, nutritional advice, exercise guidance, health management, risk identification and management, case management, etc.	RubiN created networks and support for family caregivers Reduced caregiver burden. Older adults experienced the security of caregiving.


## Multidisciplinary Team Members

36 ICT-based practice models of integrated care were delivered through the formation of multidisciplinary teams consisting mainly of clinician, practice nurse, GP, primary care physician, community nurse and social worker, with additional studies recruiting physiotherapist (38.9%), occupational therapist (27.8%), mental health practitioner (19.4%), pharmacist (27.8%) and other health care professional. Fourteen practice models also had a “case manager” role, mainly filled by nurses, community nurses, social workers or volunteers [18,19,22,25,26,27,28,30,31,32,33,34,35,38,39,43,44,49,64,69,70,71,72,73,74,81,83,84,85,86,87,88,92]. Due to the different scenarios of integrated care practice, case managers are given different responsibilities, but they are mainly responsible for case management in and out of hospital, assessing and monitoring the health and needs of older adults, organizing and coordinating care services, regularly evaluating care plans, arranging multidisciplinary meetings and providing information support. In the hospital-home-primary care facility/nursing facility transition scenario, the multidisciplinary team also includes C-TraC nurses, Link nurses, nurse coordinators, discharge nurses, GRACE support teams, etc., to coordinate and provide post-discharge support [18,24,41,42,58,71,72,81,92]. Soto-Gordoa et al [73] set up liaison nurses in hospitals to be responsible for coordinating care between different specialists during hospitalization. Weiss et al [68] recruited bilingual/bicultural social workers to respond to the needs of older adults with cognitive impairment. In addition, to guarantee the implementation of integrated care based on ICT, 16 (44.4%) practice models explicitly provided targeted training for members of the multidisciplinary team, offering a training component ranging from 2 days to 2 months [17,20,21,28,30,42,43,48,57,62,70,75,81,85,91,93]. In the Embrace model of integrated care, team member training was planned throughout the pre- and mid-program to help them work in accordance with the Embrace principles and methods [19,43,44,64,83,84]. Training for multidisciplinary team members includes various forms such as online, offline and theory + practice. For example, C-TraC nurses were required to undergo a 1-week apprenticeship with the C-TraC implementation team, in addition to a 4-week intensive training [71,72,81].

## Information and Communication Technology

The CareWell research team defined 12 ICT tools that support integrated care: electronic prescriptions, messaging between clinicians and patients, electronic health records, interconsultation, call centers, virtual conferences, personal health folders, nurse information systems, educational platforms, collaborative platforms, telemonitoring and multichannel centers [37]. We grouped the ICT support for the 36 integrated care practice models in this study into the following ten categories: digital communications(61.1%), electronic health record(33.3%), clinician and patient information system(33.3%), electronic medical record(16.7%), electronic assessment tool(13.9%), wearable monitoring device and sensor (8.3%), personal health folder(5.6%), digital educational material(5.6%), electronic prescription(2.8%), and social robot(2.8%). ICT provide easy access to ongoing monitoring, assessment, management and sharing of patient health information, communication and coordination of care among multidisciplinary team members, and documentation and monitoring of plan performance. In addition, ICT plays an important role in decision aid support, with the Embrace Integrated Care Project team embedding international functional, disability and health classification resources and official guidelines into clinical information systems to support decision-making [19,43,44,64,83,84]. The CareWell Primary Care Project has developed multidisciplinary practice guidelines for medical, nursing and social support for eight common geriatric syndromes, advance care planning practice guidelines, which are embedded in the Health and Wellbeing Information Portal for use as job aids and to promote positive dialogue between frail older adults and GP [85,86,87,88]. The World Health Organization also deliberately launched the ICOPE Handbook application to generate interventions and care plans based on the results of the intrinsic capacity assessment to help implement ICOPE in community and primary care settings [79].

## Core Elements of ICT-Based Implementation of Integrated Care

By integrating the operational approach and practice path of 36 practice models, a total of seven core elements of integrated care were identified, including single entry point, comprehensive geriatric assessment, personalized care planning, multidisciplinary case conferences, coordinated care, case management, patient empowerment. ICT was integrated and woven through the process of integrating care, facilitating horizontal and vertical integration to provide easy access to services. The seven core elements are explained below.

(1) Single entry point: A mechanism for health care providers and community-based organizations to provide services to older adults in order to increase coherence and coordination of care, often using primary care practices, health professionals as a single-entry point. Hebert et al [25,26,27] used a telephone or written referral as an entry point to services for frail older adults based on the Quebec Health Information Line, and after a needs assessment a referral to an integrated care delivery system could be made. The Walcheren Integrated Care Study team used GP as a single-entry point for older adults and their caregivers, health professionals, assessed as frail older adults with access to practice nurse visits [33].

(2) Comprehensive geriatric assessment: Multidimensional assessments of the functional health, care needs and social support of older adults and their caregivers are conducted regularly by members of the multidisciplinary team to identify participant preferences, health problems and optimization issues and generate assessment reports to guide the development of care plans. ICT was used as a vehicle for assessment tools to collect data, create reports, and share results. Delmastro [59] and Piera-Jimenez [38] used smart medical devices and sensors to collect health data on older adults. Malavasi [67] and Doyle [91] used a color-coded traffic light system in an app to customize health and well-being data reports for older adults and to send alerts and push messages when values were abnormal.

(3) Personalized care plan: The multidisciplinary team develops, updates and prioritizes the implementation of personalized goals and care plans based on the multidimensional assessment of each older person. Seven practice models of integrated care explicitly proposed the construction of evidence-based care programs based on guidelines and others, providing evidence-based recommendations from multidisciplinary teams [17,18,21,29,40,41,42,48,50,51,52,53,54,55,56,58,85,86,87,88,90]. Choi et al [90] suggested that quantitatively tailored evidence-based interventions based on ICT support are facilitators for safeguarding the fidelity of SPEC implementation. ICT provided the vehicle for care plan implementation. The CareWell Primary Care Project team stored evidence-based personalized care plans on the Health and Wellbeing Information Portal website for viewing by members of the multidisciplinary team, with a requirement to revise them at least once every six months [85,86,87,88].

(4) Multidisciplinary case conferences: Regular meetings are organized for members of the multidisciplinary team to review assessment reports on older adults, to develop, implement and adjust care plans, or to discuss complex cases and to integrate ideas from team members to provide health guidance information, in a face-to-face format. However, CareWell primary care team members communicated virtually based on a health and wellbeing information website and met every 4–8 weeks [85,86,87,88]. Rosenberg et al [80] even held daily virtual team meetings, sharing and commenting on progress notes via email. Silsand et al^18^ used a video platform for collaborative team meetings, effectively reducing the time for members to attend meetings.

(5) Coordination of care: Based on vertical and horizontal integration to coordinate health and social institutions, medical and social workers, assigning members of multidisciplinary teams to provide services to meet the needs of the elderly. Tourigny et al [28] achieved interdepartmental coordination at strategic, tactical and clinical levels by forming a joint management committee to agree on policy and direction, resource allocation. Pauly et al [93] assigned advanced practice nurses (APNs) to accompany patients and family caregivers on post-discharge visits to primary care providers to coordinate care. ICT-based features such as information sharing and virtual communication also provide facilitated channels for coordinating care.

(6) Case management: A case manager is set up and assigned to each participant in the multidisciplinary team to be responsible for planning, implementing and coordinating the care plan. Colomina et al [49] developed the Smart Adaptive Case Management (SACM) system specifically for care team members to randomly access patient files in order to coordinate professionals in different settings and to establish nurse-patient communication channels where needed. RubiN project practices have also shown that community-based care and case management play an important role in identifying, promoting and preventing family caregiver burden [69,70].

(7) Patient empowerment: Multidisciplinary team members develop an equal patient-care relationship with older adults and their caregivers, encouraging active participation in integrated care practices by stimulating patients’ inner potential, empowering them to make more decisions and choices, and sharing disease-related information and knowledge with them. The CareWell integrated care team run the empowerment program KronikOn for frail older adults and their caregivers, where primary and secondary nurses provided basic information to help patients understand their condition in order to explore and agree on the best way to care for themselves [36,37,77,78]. The Personalized Connected Care project used a mobile health self-management system for older adults and informal caregivers to access health information and communicate fully with the care team [49]. The involvement of older adults played an important role in promoting ICOPE implementation.

## Service Contents and Practice Effects of ICT-Based Integrated Care

36 practice models integrated medical and social resources in order to provide a full range of care services. In addition to basic services such as professional medical care (medical treatment, nursing, rehabilitation, psychological counselling, nutritional support, medication and exercise guidance, etc.), primary health care, social support (community resources, volunteer support) and home care, they also provide telehealth (including telemedicine, telemonitoring, telecare and teleconsultation) (30.6%), day care (13.9%), home help (11.1%), caregiver support (8.3%), end-of-life care (8.3%), et al. Colomina et al [49] also customized virtual coaches with automatic feedback for older adults based on health status reports to provide a lively and personalized health education service.

The length of ICT-based integrated care services ranged from 3 to 36 months, with high program practice feasibility and satisfaction, improving coordination and collaboration between health and social workers. Evaluated from the demand side of the service, the model was effective in improving the quality of life of older adults [24,30,39,49,64,74,77,78,89], enhancing chronic disease self-management [75], reducing the number of unplanned emergency room and hospital visits [24,25,26,27,28,36,37,40,41,46,47,48,49,51,71,73,75,80,81,92], preventing physical, cognitive and social decline [17,25,26,27,28,45,48,82,83], and reducing the burden on caregivers [25,28,31,68,70,75,82]. From a supply-side evaluation, ICT-supported integrated care significantly improved the quality of care [17,22,41,57,68,69,76], increased access to primary health care and community resources for older adults [18,26,36,37,46,47,65,68,76,77,78,89], and the 11 practice models were cost-effective, saving money on medical and care costs [29,38,40,42,49,51,71,72,75,76,77,78,81,89,92,93]. However, eight practice models still failed to improve quality of life, functional capacity and healthcare resource utilization among older adults [19,28,30,33,38,47,57,74,85,86], and four practice models had no net monetary benefit due to their own high operating costs [34,35,44,87], and further comparative analysis is urgently needed to explore optimal integrated care pathways.

## Hindrances and Facilitators of ICT-Based Practice of Integrated Care

ICT plays a key role in all aspects of integrated care, including community resource and policy, health system, delivery system, self-management support, decision support and clinical information system, but numerous factors still hinder the use of eHealth technology. Six studies described barriers to practice ICT-based integrated care [20,23,59,61,79,91], grouped under four themes: demand-side factors (fear and lack of confidence in applying IT, lack of skills of patients, lack of trust in the accuracy of smart monitoring devices), supply-side factors (lack of skills of providers, resistance to innovative applications of IT, lack of human resources), technical factors (inadequate ICT infrastructure, poor compatibility between eHealth tools, inadequate ICT technical support, use of devices and applications process complexity, privacy/security issues), systemic factors (inadequate legislative framework, inadequate funding, uncertainty of cost effectiveness). However, Kastner [20], Vestjens [22] and Valaitis [66] identified adequate human resources, multidisciplinary member involvement, training and regular communication, continuous review and feedback, development of procedures and/or protocols to support team processes, sustainability infrastructure, facilitated ICT systems, clinical leadership involvement, and structured funding as key to guaranteeing the sustainable spread of ICT-supported integrated care models. The active participation of older adults, training of providers, and digital integration of health information were also identified as important facilitators by the World Health Organization in its report on the work of ICOPE Practice [79].

## Discussion

The ICT-based integrated care model follows the core elements of single entry point, comprehensive geriatric assessment, personalized care planning, multidisciplinary case conferences, coordinated care, case management, and patient empowerment to provide the services needed for older adults, which preliminary practice has shown to improve physical and mental health and quality of care for older adults, save health care resources, and enhance primary care and community resource utilization, but there is heterogeneity in practice outcomes and numerous influencing factors remain at the demand-side, supply-side, technology, and system levels.

At the demand-side level, the main targets of ICT-based integrated care services are frail older adults (30.6%), older adults with physical or cognitive impairments (16.7%) and older adults with multiple morbidities (13.9%). The increasing prevalence of frailty with age, the consequent deterioration in physical, cognitive, social and psychological conditions, the increasing complexity of health and social care needs, and research showing that multiple morbidities were associated with increased unmet needs, health care utilization and reduced perceived health status and quality of life [94], have a greater preference for integrated care services involving multiple supply actors. Older patients themselves have expressed a desire for accessible, efficient and coordinated care that meets their needs and preferences, while keeping in mind their rights and safety [95]. Islam et al [96] constructed a “Holistic Continuum of Patient Care” program specifically for frail older patients to provide integrated care, and their practice addressed the issue of multiple morbidity.

However, the ICT-based integrated care was hampered by older adults’ fear, lack of confidence and skills of using information technology (IT). This may be related to the varying degrees of ‘technophobia’ among older adults [97], resulting in lower use and acceptance of ICT and indirectly influenced by cognitive closure, resulting in poorer e-health readiness among older adults [98]. This suggests that researchers could subsequently develop training programs and ‘age-friendly’ information platforms to enhance the acceptance of information technology among older adults, guided by the causes of their ‘technophobia’. In addition, the lack of trust in the accuracy of smart monitoring devices among older adults is also a deterrent to participation. However, with the widespread use of IT in healthcare, internet of things(IoT) technologies such as sensors and wearable devices have been identified as a more suitable vehicle for health monitoring and comprehensive geriatric assessment [99], which can effectively improve the ease of data collection and sharing, and the potential benefits of telemonitoring in reducing disease progression and hospitalization in older adults with long-term conditions [100]. The Government can take the lead in placing smart monitoring devices in primary care facilities, providing free application experience for older adults and promoting application on the basis of gaining trust.

At the supply-side level, the multidisciplinary team of mainly health professionals, primary care workers and social workers providing services is an important internal driver of integrated care. The involvement of multidisciplinary members helps to meet the complex care needs of older adults and the organization of regular multidisciplinary meetings can help to define the scope of action of members, coordinate care services and is important to improve support for patients and their families, with GPs playing an important role in the successful delivery of care for older adults [101]. In addition, GPs and case managers are often seen as a single point of entry to integrated care. Having a single point of entry facilitates the referral of older adults to appropriate social and/or primary care institutions, it’s important for the integration of services and the standardization of the needs assessment process [102], and the single entry system ensures a sufficient volume of patients for financial stability and efficient operation, which helps to ensure that social resources are based on medical needs [103]. It is suggested that researchers could recruit multidisciplinary members to form a service team using the results of a comprehensive assessment of older adults as a guide, making full use of the GP as a single-entry point.

The lack of human resources for services, the lack of skills of providers and resistance to innovative applications of IT are important factors that hinder the implementation of ICT-based integrated care. In 2018, there was a global shortage of approximately 6 million human resources for nurses and a projected demand shortage of 5.7 million nurses remains in 2030, a phenomenon that is particularly evident in the COVID-19 environment, severely impacting the matching of supply and demand for integrated care services [104]. In addition, the IT usage behavior of the multidisciplinary team members, as users of ICT, has a direct impact on the planned implementation and the quality of integrated care. Hector et al. [105] showed that health care assistants perceive ICT to be unhelpful, time-consuming to adopt, burdensome or increasing in workload, which all contributed to resistance to the use of ICT by service providers. This suggests that clinical managers should streamline the process of operating ICT platforms and provide targeted training on ICT applications to strengthen nurses’ attitudes and competencies in the use of IT. Healthcare professionals considered that helping to raise awareness of e-health expertise by exposing healthcare professionals to relevant IT solutions and medical technology is the best training initiative to improve their IT skills [106].

Information and communication technologies commonly used in integrated care models included digital communication (61.1%), electronic health records (33.3%), and clinician and patient information systems (33.3%), similar to the results of Melchiorre et al [60] who analyzed the use of e-health tools in European integrated care projects for older adults with multiple morbidities. It can be attributed to that digital communication facilitates timely communication between multidisciplinary teams and with patients, that electronic health records enable the collection and sharing of patient information, and that information systems provide portals to team members and patients in order to empower patients and promote their active participation in the implementation of integrated care [107]. Electronic prescriptions (2.8%) and social robots (2.8%) were less commonly used, which may be related to the fact that ICT-based prescriptions are less frequently issued and transmitted in integrated care services, and that pharmacists make up only 27.8% of the multidisciplinary team members, which, combined with controls on the cost of care, has somewhat influenced the use of e-prescribing and artificially intelligent bots. However, both older patients and their informal caregivers placed a high value on both robotic-assisted and non-robotic-assisted technology as a care pathway [108]. In addition, electronic prescription can reduce medication errors and adverse drug reactions, improve prescription safety [109], and pharmacists are willing to participate in electronic prescription systems [110]. This suggests that researchers could add e-prescribing services to integrated care and could also provide spiritual comfort services with the help of robots.

At the technical level, inadequate ICT infrastructure, limited functionality and complex processes for using technology, poor compatibility between e-health tools and privacy/security issues are impediments to integrated care implementation. MARTONO et al. [111]found that poor ICT system quality and information quality reduces users’ perceived usefulness and perceived ease of use. Based on technology acceptance model analysis, perceived usefulness and ease of use affect users’ behavioral intentions. In addition, collaborative care between multidisciplinary teams requires the sharing of patient data, and the main challenges to data transfer are privacy and security issues. The study found that users’ perceived privacy and perceived security affect their willingness to continue ICT adoption [112]. Ogal et al. [113] also identified interoperability and compatibility of ICT systems, and privacy issues as major barriers to sharing healthcare records, which can affect smooth communication and coordination of care between multidisciplinary teams and reduce stakeholder trust and user engagement. User engagement affects their perceived usefulness, perceived ease of use and behavioral intentions [114]. This suggests that researchers can design comprehensive, convenient and interoperable platforms for integrated care services based on the technical functional requirements of both the supply and demand sides of the service.

At the system level, there are still impediments to the operation of ICT-based integrated care, such as inadequate legislative frameworks, insufficient funding and uncertainty about cost-effectiveness. For example, the England has had a succession of policies in place since 2010 to encourage the integration of health and social care, but the layering of numerous policy initiatives has affected the establishment of integrated relationships and the chronic underfunding of social care has led to significant workforce challenges [115]. In addition, inadequate financial support and weak cost-effectiveness could discourage the allocation of funds to individuals, hospitals and departments, and thus fail to incentivize the integration of care [116]. The German Federal Government has launched the Healthcare Innovation Fund, which provides €200 million per year from 2020–2024 to support the development and diffusion of integrated healthcare and to stimulate relevant insurance companies to support the development of new models of integrated care [117]. Policies introduced by the US and state governments have initiated health funding to provide financial support for social care [118]. Stokes et al [119] stimulated more integrated activities by pooling health and social care funding. Four practice models in this study showed no net monetary benefit and somewhat reduced incentives for multidisciplinary team members, older adults, and their caregivers to participate in integrated care, possibly because recruiting multidisciplinary team members increases labor cost expenditures, while short-term interventions do not improve health outcomes for frail or multiply chronically ill older adults, and early implementation of the intervention’s aggressive practices may increase older adults’ use of services and informal care, indirectly increasing the cost of interventions. This suggests that governments should take the lead in incentivizing multiple sources of financing to provide appropriate services based on matching supply and demand to reduce the cost of interventions and protect economic benefits.

## Conclusion

The ICT-based integrated care model used digital communication, electronic health record, clinician and patient information systems as vehicles to form multidisciplinary teams to provide diversified services by vertical and horizontal integration of health and social care institutions, combining seven core elements of single entry point, comprehensive geriatric assessment, personalized care planning, multidisciplinary case conferences, coordinated care, case management and patient empowerment, which met the needs of both service providers and demanders to some extent. However, there is still heterogeneity in their practice effects and the team will conduct further systematic review to assess the actual effect of ICT-based implementation of integrated care through a rigorous quality evaluation of the literature and consolidation of results. Moreover, fewer included studies focused on barriers and facilitators of ICT-based implementation of integrated care, and the combined evidence may not be convincing; researchers could use qualitative research to gain insight into the current state of local practice and key elements to facilitate successful implementation of ICT-based integrated care before formal intervention.
